# Transcatheter Aortic Valve Replacement as a Bridge to Minimally Invasive Endoscopic Mitral Valve Surgery in Elderly Patients

**DOI:** 10.3390/jcm13020471

**Published:** 2024-01-15

**Authors:** Tamer Owais, Osama Bisht, Emre Polat, Noureldin Abdelmoteleb, Mohammad El Garhy, Phillip Lauten, Thomas Kuntze, Evaldas Girdauskas

**Affiliations:** 1 Department of Cardiac Surgery, University Hospital Augsburg, 86156 Augsburg, Germany; tamer.owais@uk-augsburg.de (T.O.); noureldin.abdelmoteleb@uk-augsburg.de (N.A.); evaldas.girdauskas@uk-augsburg.de (E.G.); 2 Department of Cardiothoracic Surgery, Cairo University, Giza P.O. Box 12613, Egypt; 3 Department of Cardiology and Angiology, Regiomed Klinikum Coburg, 3396450 Coburg, Germany; 4 Department of Cardiology, Helios Clinic Erfurt, 99089 Erfurt, Germany; mohammad.elgarhy@helios-gesundheit.de; 5 Department of Cardiology, Heart Center, Zentralklinik Bad Berka, Robert-Koch-Allee 9, 99437 Bad Berka, Germany; philipp.lauten@zentraklinik.de (P.L.); thomas.kuntze@zentralklinik.de (T.K.)

**Keywords:** TAVI, endoscopic mitral valve repair, minimally invasive cardiac surgery

## Abstract

In this bicentric study, we report the outcomes of combined transcatheter aortic valve replacement combined with minimally invasive mitral valve surgery. We included a cohort of six patients (79.6 ± 3.2 years, 83% women) with high-risk profiles and deemed to be non-operable with combined mitral and aortic valvular disease. All patients had unsuitable anatomies for transcatheter mitral valve edge-to-edge repair (TEER). Moreover, most of the patients (5/6) suffered a combined aortic valve lesion, which complicates the efficiency of cardioplegia in the case of CBP through minimally invasive incisions. The first stage was implanting a TAVI valve to achieve aortic valve competency and hence facilitate the infusion of cardioplegia after clamping the aorta during endoscopic mitral valve surgery. After one week, we performed the minimally invasive mitral valve repair. Most patients (n = 5; 83%) underwent successful endoscopic mitral valve repair. Intraoperatively, the mean ischemic time was 42 min, and the total bypass time was 72 min. Postoperatively, the mean intubation time was 0 h. Postoperative complications included reoperation for bleeding in one patient (16.7%) and a new heart block requiring pacemaker implantation in one patient (16.7%). There was neither in-hospital mortality nor 1-year mortality.

## 1. Introduction

Despite the progressive rise in the number of cases in need of mitral surgery and improved outcomes, the cohort of patients with concomitant regurgitant aortic valves remains, particularly, a high intraoperative challenge. Ensuring efficient myocardial protection and sufficient cardioplegia in an incompetent aortic valve concerns all surgeons to establish proper outcomes. Here, we focus on a confined group of patients with mitral valve pathology who are relatively old with high operative risk, non-clippable mitral pathology, and a concomitantly incompetent aortic valve. It is worth noting that the option of TAVI in MAC was not offered despite being a specialized center in such procedures because of a lack of circumferential calcification in all of our patients. The options offered are very limited. Full sternotomy with combined mitral and aortic valve operation will be a burden on such high-risk patients. Our idea involved a staged procedure to produce a competent aortic valve using our experience in the TAVI field and the huge development in TAVI valve industry, then carrying out an endoscopic video-assisted mitral valve operation and directly extubating the patient either in the operating room or directly thereafter, then transferring the patient to the intermediate care. In conventional mitral valve surgery, multiple reports state 30-day mortality ranging between 6.3 and 15% for elective cases and 17.8% for emergency cases [1,2]. Mitral valve surgery in elderly inoperable patients is a high-risk procedure with surgical and anesthesiological challenges, with reported considerable operative and postoperative morbidity of up to 8% bleeding and prolonged intubation longer than 48 h in 14.4% with high consumption of inotropes in the ICU postoperatively (3.4%).

The fact that several patients from the high-risk group with mitral pathology, which is not amenable to the clip, are usually directed in a conservative way aroused our interest in implementing our endoscopic procedures to the benefit of this domain of patients. Together with the era of enhanced recovery after surgery (ERAS) concept in cardiac surgery and the fast-track strategy in TAVI procedures, we were able to minimize the burden of surgery on the patient and his family concerning complications and duration of hospital stay.

Enhanced recovery after surgery (ERAS) is a multimodal, intersectoral, transdisciplinary care improvement initiative to promote fast and complete recovery of patients undergoing surgery throughout their entire perioperative journey. It is based on the reduction in surgical trauma.

The main aim of minimally invasive techniques and ultra-fast-track anesthesia with on-table extubation or early extubation <6 h postoperatively is focusing on early mobilization. Interest in the application of ERAS to cardiac surgery has grown significantly over the last few years.

These programs aim to reduce postoperative complications, reduce hospital stays, and return patients to their normal functional status as early as possible. Initial clinical data after the first year of a hospital-wide ERAS cardiac program were associated with significantly improved perioperative outcomes. Nowadays, it is believed that this value-based approach to cardiac surgery can result in earlier recovery, reduced opioid use, LOS, cost, and increased patient/staff satisfaction [1].

The movement towards minimalist approaches (MAs) and fast-track (FT TAVI) protocols for patients undergoing TAVI has gained appreciable momentum in recent years, not only due to concerns about optimal resource utilization but also to decrease the perioperative complications. Our (FT-TAVI) protocol includes avoidance of general anesthesia, performing the procedure only under conscious sedation with percutaneous access, use of low-dose or short-acting opioids, short rapid-pacing phase, low-dose contrast solution, and earlier transfer to the normal ward on a telemetry bed, hence achieving discharge at home on the fifth postoperative day [2].

The combination of both concepts (ERAS and fast track) in patients who are suffering not only from severe mitral regurge, which is graded as non-clippable mitral valves and at the same time non-suitable for sternotomy, but also having sclerotic aortic valves with mild-to-moderate stenosis, and at least moderate regurge would change the medical situation for those patients from inoperable patients needing palliative care to operable patients with the minimalist invasive procedures, least perioperative complications, and best outcome postoperative. The combination of the ERAS project and fast-track TAVI project may play a role in changing the decision of the cardiac surgeon for those patients.

## 2. Materials and Methods

We conducted an observational study for high-risk patients (EuroScore II > 10%) with combined mitral and aortic pathologies who underwent either minimally invasive therapy of both valves (6 patients treated with combined TAVI and minimally invasive mitral surgery) vs. combined open mitral and aortic valve surgery (22 patients). All the patients included in the study group were treated during the period between September 2019 and March 2022 in University Clinic Augsburg and Central Clinic Bad Berka.

The two involved participating centers used standardized preoperative data collection, operative protocols, and techniques, as well as postoperative variables with clinical outcomes from the index hospitalization until one-year follow-up. We demonstrated all demographical data, including age, gender, diabetes type II, carotid stenosis, chronic renal insufficiency, chronic obstructive pulmonary disease, degree and morphology of mitral and aortic valve pathology, degree ejection fraction, pulmonary hypertension, NYHA class (New York Heart Association), EuroSCORE II, and atrial fibrillation. Preoperatively, all patients were discussed in a multidisciplinary structural heart team consisting of structural heart cardiologists, a cardiothoracic surgeon (who worked in the two centers), and an anesthesiologist. Routinely, preoperative cardiac catheterization, transthoracic echocardiogram (TTE), lung function tests, laboratory investigations, and cardiac computed tomography (CT) scans were conducted. Special consideration was taken to determine the detailed morphology of the mitral and aortic valve, coaptation defect, and presence of prolapse, leaflet displacement, and etiology as well. Among those high-surgical-risk cohorts, patients with morphology amenable to clipping were excluded and hence scheduled for Mitraclip in elective form. We included only patients with non-clippable mitral morphology and aortic valve sclerosis with a degree of aortic regurge as well. Classically, edge-to-edge repair has been adopted in patients with central pathology and non-calcified leaflets; this has been adapted in the EVERST-Trial. Ideal candidates are patients with pathology in A2/P2 segments, adequate mitral valve area (4.0 cm^−2^), non-calcified leaflets, adequate leaflet length > 10 mm, flail gap less than 10 mm, and elevated mean gradient > 5 mmHg. Those criteria have been adapted from the EVERST-Trial [3]. Patients who do not fit those criteria may still be eligible for Mitral-TEER [4] with favorable outcome, which has been demonstrated in EXPAND-Registry [5]. In our study, every patient underwent a comprehensive assessment including 2D and 3D TEE; the likelihood of success was individually assessed by a team consisting of an experienced interventional cardiologist, an imaging specialist, and a cardiac surgeon. The decision was based on valve morphology. Severe mitral annular calcification, mitral stenosis with area less than 2.5 cm^−2^, and endocarditis were considered absolute contraindication. Those patients were scheduled for elective TAVI after fulfilling the preoperative TAVI workup (CT and echo). All operations took place in the hybrid room with a heart–lung machine on standby and under local anesthesia. One week later, the patients were scheduled for the totally endoscopic mitral valve procedure in the surgical operation theatre.

For the MIC procedures, the patient was positioned supine with the right hemithorax tilted up 30 degrees using a sandbag. A right anterolateral mini-thoracotomy was performed through a 4–5 cm peri-areolar incision, and the soft tissues were retracted. Additionally, pericardial stay sutures of the lower pericardial edge were sutured to the soft tissue retractor. Another port in the fourth intercostal space mid-axillary line was used for the insertion of a 3D endoscope. Arterial and venous cannulation for extracorporeal circulation was performed using the Seldinger technique and transesophageal guidance. Body temperature is maintained at around 34 °C. A third port in the second right intercostal space in the anterior axillary line was used for cross-clamping the ascending aorta, and due to competency of implanted TAVI prosthesis, antegrade Del Nido cardioplegia was notably delivered antegrade into the aortic root for efficient myocardial protection.

The left atrium is accessed through a left atrial incision, an atrial retractor was applied, and a conventional mitral valve procedure was performed. The hemodynamically stable patient was then extubated successfully in the operating room and transferred to ICU for one night and discharged at home after five days without any perioperative events.

Perioperative data were collected, including previously implanted aortic bioprosthesis, mitral annular ring or prosthesis, operative time, cross-clamp time, need for inotropes, and transesophageal echocardiogram (TEE).

Postoperative data including hospital stay, intubation time, NYHA class, and other parameters were collected as well.

## 3. Statistical Analysis and Results

**Figure A1:**
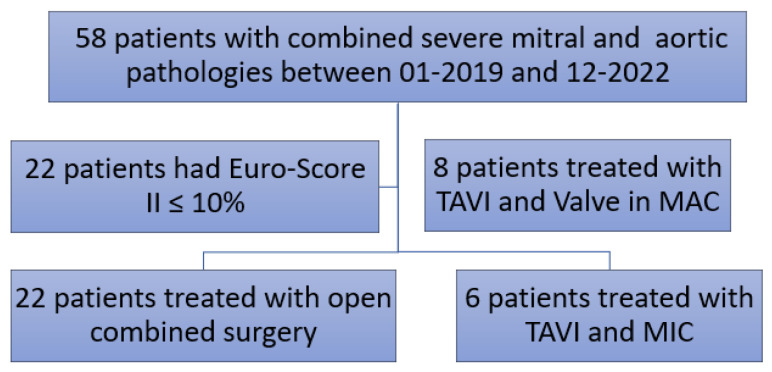
Study flowchart.

From 44 consecutive patients who underwent double mitral and aortic valve surgery at Central Hospital Bad Berka, Germany, and Augsburg University Hospital, Germany, between September 2019 and March 2022, we identified 22 high-risk patients (control group). The study design and concept is highlighted in Appendix A, Figure A1. We compared this control group with the study group, six patients treated with TAVR followed by MIC procedure. These patients were identified from our hospital’s archive. Continuous variables are expressed as means and standard deviations and were tested for statistical significance with Fisher’s exact test to detect the difference between the two groups. Categorical data are expressed as percentages and were compared by chi-square statistics. Statistical analyses were performed with SPSS (version 24.0; IBM Corporation, Armonk, NY, USA). A two-sided *p* < 0.05 was considered statistically significant.

The baseline characteristics of our cohort are summarized in Table 1. The average age was 79.6 ± 3.2 years with higher prevalence (83%) of female sex. In the control group, female gender was prevalent in 77% with mean age 78.1 ± 1.2 years. In the control group, three patients had a pacemaker preoperatively versus none in the study group. It is worth mentioning that all six patients in this group received TAVI with balloon-expandable valves with a higher implantation level, and hence, none of them needed a permanent pacemaker postoperatively. Nine patients in the control group were chronic AF patients and on warfarin therapy, which was stopped 5 days preoperatively until an INR between 1 and 1.3 was reached. This may explain the 27% bleeding rate among this group together with the relatively longer bypass time and longer hypothermia, which may have been affected by the coagulation cascade in those patients. Six patients in the control group were COPD patients on long-standing salbutamol therapy, and two of them were on corticosteroid regimen, and this in addition to the sternotomy wound and longer operative time may explain the longer intubation time postoperatively and the incidence of sternal wound dehiscence or infection as well. 

Intraoperative variables are shown in Table 2. The majority of patients in the study group (n = 5; 83%) underwent successful endoscopic mitral valve repair, while intraoperatively, the mean ischemic time was 42 min, total bypass time was 72 min, and all patients regained sinus rhythm immediately after declamping the aorta. These results compare to 108 min of ischemia and 159 min on bypass in the control group.

Postoperative data were collected and presented in Table 2 as well. The mean postoperative intubation time was 0 h in the study group versus 7 h in the control group. Postoperative complications in the study group included reoperation for bleeding in one patient (16.7%) and new heart block requiring pacemaker implantation in one patient (16.7%). There was neither in-hospital mortality nor 1-year mortality. At the latest follow-up, four of the six patients were in NYHA class I, one patient in NYHA class II, and one patient was in NYHA III due to newly developed severe tricuspid valve regurge. Echocardiographically, 83% of patients (5/6) showed improvement in the left ventricular ejection fraction at follow-up, with no evidence of paravalvular leak, infective endocarditis, or progressive valve degeneration. 

## 4. Discussion

Multiple and mixed VHD are extremely common diseases. Multiple VHD, defined as at least two moderate VHDs, was seen in the Euro Heart Survey in 20% of individuals with native VHD and in 17% of those undergoing intervention [6]. The combination of valvular aortic stenosis and mitral regurgitation is only second to the concomitant affection of both AV valves, affecting approximately 10% of patients undergoing surgery for VHD [7]. Paradoxically, there are few recommendation guidelines regarding these patients. The establishment of specialized cardiac valve centers with a multidisciplinary heart team facilitates clinical decision making as a combination of multiple surgical and transcatheter techniques are required. Elderly patients with multiple comorbidities constitute an important subgroup of this patient population, thus providing treatment with a minimal amount of surgical trauma is crucial to reduce the amount of morbidity and eventually mortality associated with treatment. 

There are limited recommendation guidelines for patients with moderate-to-severe aortic stenosis in combination with severe primary mitral regurgitation. The ACC/AHA guidelines recommend treatment of the aortic valve and consider TEER as the primary treatment modality if the patient is not a candidate for surgery [8].

Transcatheter aortic valve replacement has been widely adopted as less invasive treatment for valvular aortic valve stenosis in high- and intermediate-risk groups with comparable results to surgical aortic valve replacement (SAVR) on the midterm follow-up. The current ESC/EACTS guidelines recommend TAVI as the preferred treatment approach for patients above 75 years of age, and to patients who are unsuitable for surgery (STS-Score or EuroScore II > 8%). However, the development of transcatheter mitral valve technologies has been lagging behind the aortic counterpart. This could be explained by the fact that surgical mitral valve repair involves multiple techniques like undersized annuloplasty rings, wedge resections, neo-chord implants, and leaflet augmentation, often in combination depending on the pathology and surgical expertise. On the other hand, transcatheter edge-to-edge repair is the only widely available option for transcatheter mitral valve repair. This treatment involves approximation of both leaflets and restoring central coaptation of the mitral valve, which has been inspired by Alfieri stich repair. Many experienced high-volume operators reported excellent real-world outcomes in treating complex mitral pathologies [5]; however, the treatment is far from becoming standard of care. 

The interaction between multiple valvular heart disease is characterized by complex hemodynamics and is poorly studied. The simultaneous presence of severe mitral regurgitation leads to lower forward stroke volume, thus underestimating the severity of aortic valvular stenosis [9,10]. Therefore, the gradients across the aortic valve may be falsely reduced, producing a paradoxical low-flow, low-gradient aortic valvular stenosis. Thus, our cohort of patients underwent a thorough assessment using cardiac computerized tomography. The average calcium score of our patients was 1430 in females and 2150 in males, which is above the cut-off of 1200 HU for females and 2000 for males, considered to offer a high discriminatory value for serve valvular aortic valve stenosis [11]. After reviewing the echocardiographic and CT findings of our cohort, the heart team felt that the aortic valve pathology needed to be addressed. Moreover, the presence of at least moderate calcification allowed safe anchoring of the valve with minimal risk for embolization and adequate sealing without significant paravalvular leak. 

Although improvement of the mitral regurgitation after the TAVI procedure has been reported, some authors reported less favorable results of edge-to-edge repairs in patients with previous TAVI implants [7]. In the study by Patzelt et al. [12], there were fewer patients with optimal outcome after TEER. Thus, combining the TAVI with more favorable results of endoscopic mitral surgery appears to be an attractive alternative.

Current surgical techniques involve double-valve replacement and/or involve full sternotomy with central cannulation for extracorporeal circulation. The outcomes of those approached have been historically associated with high morbidity and mortality. In the study by Eggers et al. [13], the 30-day mortality was high as 7%. Another study reported a mortality of 9.7% for patients undergoing combined mitral and aortic valve surgery [14]. The major sources of morbidity and mortality include prolonged ventilation and ICU stay, postoperative renal failure and postoperative bleeding, and stroke.

The concept of the current study is to harness the benefits of the TAVI procedure, which has very good short and intermediate outcomes, combined with benefits of the endoscopic mitral valve repair to achieve an excellent repair in wide array of pathologies and great long-term durability in a hybrid approach aiming to reduce the morbidity and allowing the heart team to offer hybrid treatments for otherwise inoperable patients.

### Technical Consideration

Preprocedural planning of TF-TAVI procedure is beyond the scope of this paper and has been described elsewhere. Concerning the choice of the THV for the TAVI procedure, we preferred the balloon-expandable Edward valve prosthesis for our patients because of several reasons. The S3 valve has a short frame when deployed, which allows the surgeon to clamp the ascending aorta with minimal risk of interaction with the valve frame, thus minimizing the risk of deformation to the valve frame. This short frame also has a minimal extension in the LVOT, which gives unrestricted ability to place sutures in the anterior mitral leaflet, in particular the A1 and A2 regions. A meticulous deployment technique with so-called 90/10 deployment is essential; this means that only 10% of the valve should be encroaching on the LVOT. A high deployment is necessary to allow unobstructed surgical manipulation of the mitral valve. In a retrospective analysis, Meyer et al. demonstrated a reduction in the mitral anteroposterior diameter and mitral valve area after TAVI; the reduction in anteroposterior diameter was significantly more pronounced in case of the BEV [15]. This may explain the more pronounced reduction in the mitral valve severity after BEV compared to SEV. To further test the validity of this observation, a computerized simulation of different TAVI prosthesis and different implantation depth were performed to observe the changes in the aorto-mitral continuity. When comparing SEV vs. balloon-expandable valve (BEV) performance at an optimal implantation height, the SEV gave a higher regurgitant volume than the pre-TAVI model (40.49 vs. 37.59 mL), while the BEV model gave the lowest regurgitant volume (33.84 vs. 37.59 mL) [16]. Further work is needed to study the effect of different TAVI prosthesis on the aorto-mitral curtain, annular diameter and area, and aorto-mitral angle, which are crucial for planning and executing mitral valve repairs.

Regarding the surgical planning, several points should be taken into consideration. The site of cannulation: in our study, all the patients received a peripheral cannulation utilizing the femoral vessel. Planning the puncture site was facilitated by carefully evaluating the preprocedural TAVI-CT. In case of unsuitable femoral access, direct axillary cannulation is a valid alternative. The myocardial protection strategy in our cohort involved non-selective antegrade Del Nido cardioplegia after clamping the aorta. The incision was a right mini-thoracotomy with incision between 4 and 6 cm with excellent cosmetic results. 

Compared to standard surgical exposure, our technique offers several advantages, which include:

1. Avoiding median sternotomy: This leads to lower pain scores postoperatively with easier mobilization and recovery after surgery; this has been demonstrated in the study performed by Huang et al. [16].
2. Lower ischemic time when compared to double-valve surgery: In our study, the mean ischemic time was 42 min, which is lower than the numbers reported in double-valve surgery. Husso et al. [17] stated that the mean aortic cross-clamp time was 161 min, and the mean perfusion time was 199 min. Thus, using our approach allowed the surgeon to remain under 100 min of total CBP time. No case in our cohort required more than 72 min. Prolonged CBP time especially over 180 min directly correlates with mortality even after adjusting for EuroSCORE II, postoperative complications, prolonged ICU stay, and prolonged mechanical ventilation [18].
3. Lower blood transfusion rate: In our patient population, the average amount of blood units received was one pack of RBCs, which is well below the average for double-valve surgery. This could be logically explained through avoiding sternotomy wounds in our cohort and hence decreasing the amount of blood loss through minimally invasive endoscopic approaches.
4. Lower mechanical ventilation and ICU stay: In our population, there was no need for prolonged mechanical ventilation. All patients were successfully extubated with average duration of mechanical ventilation of 3.2 h. It is well known that prolonged mechanical ventilation after cardiac surgery correlates with adverse outcomes including mortality. It was reported that patients who have prolonged mechanical ventilation have an ICU mortality as high as 44.3% vs. a mortality of 3.1% for patients who could be successfully extubated [19].
5. Regarding the postoperative adverse outcome: All our patient had uneventful recovery except one patient who developed a significant postoperative bleeding. This patient had the endoscopic MIMV under therapeutic anticoagulation and antiplatelet treatment, because of recent cardioversion for atrial fibrillation. Due to high drainage postoperatively, we had to stop the anticoagulation therapy after exclusion of the LAA-Thrombus per TEE in the ICU 3 h after the operation. This led to cessation of the bleeding.

The long-term durability of the combined TAVI/MVIVR is considered to be excellent. Recent studies have reported excellent durability of the THV at 5–8 years with structural failure rates between 2.4% and 4.5% [20]. In one in vitro study which compared the durability of Sapien Edwards THV to its surgical counterpart, there was excellent durability at a one-billion-use cycle, which is equivalent to 25 years of use with a failure rate around 2.5% [21,22]. Even if structural valve deterioration occurs, the TAVI-in-TAVI procedure is still possible with excellent outcomes. As regarding structural deterioration on the mitral side, both percutaneous valve-in-ring and valve-in-valve utilizing the transseptal approach with Sapien platform are excellent alternatives to redo surgery. Recently, the food and drug administration approved the commander system and Sapien valve to be implanted via the transseptal approach. 

## Figures and Tables

**Table 1:** Baseline characteristics.

**Variables**	**Study Group (n = 6)**	**Control Group** **(n = 22)**	***p*** **-Value**
**Age** (years)	79.6 ± 3.2	78.1 ± 1.2	0.30
Female	5	17	0.40
COPD	1	6	0.30
CRF > II	2	8	NA
DM II	3	15	0.20
Carotid stenosis			
uni-lateral	1	9	0.50
Bi-lateral	1	2	0.20
Pacemaker	1	3	0.40
Pulmonary hypertension	3	15	0.30
Previous myocardial infarction	1	4	0.20
LVEF 50–60%	5	18	0.45
Atrial fibrillation	1	9	0.55
NYHA			
I	0	0	0
II	0	4	0.40
III	6	18	0.55
Euroscore Log.	17.9 ± 13.3	16.8 ± 12.1	0.10
Aortic valve			
- **Mean gradient ≥ 40 mmHg**	5	12	0.35
- **Mean gradient < 40 mmHg**	1	10	0.55
- **AVA < 1.0 cm^2^**	4	14	0.25
- **AVA 1.0–1.5 cm^2^**	1	8	0.25
- **AVA > 2.0 cm^2^**	1	15	0.45
- **Calcification score > 1200**	4	9	0.23
Mitral valve			
- **MR ≥ III**	5	7	0.31
- **MS ≥ III**	1	15	0.12
- **Significant prolapse**	5	5	0

Data presented as mean ± standard deviation or number (percentage). COPD: chronic obstructive pulmonary disease; CRF: chronic renal failure; DM: diabetes mellitus; LVEF: left ventricular ejection fraction; NYHA: New York Heart Association; AVA: aortic valve area.

**Table 2:** Operative results and postoperative endpoints.

Variable	Study Group (n = 6)	Control Group (n = 22)	*p*-Value
Edwards sapien 3			NA
23 mm	3	0	
26 mm	2	0	
29 mm	1	0	
Edwards perimount (aortic)			NA
21	0	2	
23	0	14	
25	0	8	
Mitral valve			
- **Replacement**	1	15	0.05
- **Repair (loop + ring)**	5	7	0.30
Bypasstime (minutes)	72	159	0.01
Ischemic time (minutes)	48	108	0.01
Extubation in OP	6	0	0.01
Need for catecholamines	1	18	0.03
Mean postoperative hospital stay	5	17	0.01
Mean ICU stay	0	3	0.2
Need for pacemaker	1	4	0.2
bleeding	1	6	0.15
Postoperative SR	5	9	0.3
Conversion to sternotomy	0	22	NA
Pericardial tamponade	0	6	0.01
Annular rupture	0	0	0
Postoperative endocarditis	0	3	0.25
PVL > I	0	1	0.20
Postoperative stroke	0	3	0.15
Wound dehiscence	0	4	0.25
LVEF > 60%	5	17	0.30
NYHA			
I	4	16	0.01
II	1	4	0.15
III	1	2	0.1
Del Nido cardioplegia (liters)	1.3	2.9	0.3
Number of transfused packed RBCS	1	4	0.21

Data presented as mean ± standard deviation or number (percentage). OR: operating room; SR: sinus rhythm; pVL: paravalvular leakage.

## Data Availability

The data used in this study are available upon reasonable request.

## Limitation

Due to the non-randomized nature of this study, the results cannot be generalized to the whole population with double-valve pathology. The presence of an experienced minimally invasive heart surgeon is crucial to the success of such procedures.
